# Circular RNAs regulate parental gene expression: A new direction for molecular oncology research

**DOI:** 10.3389/fonc.2022.947775

**Published:** 2022-08-25

**Authors:** Haicun Wang, Xin Gao, Shaobo Yu, Weina Wang, Guanglin Liu, Xingming Jiang, Dongsheng Sun

**Affiliations:** ^1^ General Surgery Department, The 2nd Affiliated Hospital of Harbin Medical University, Harbin, China; ^2^ Department of Anesthesiology, The 2nd Affiliated Hospital of Harbin Medical University, Harbin, China

**Keywords:** circRNAs, parental gene, regulatory mechanism, tumors, molecular oncology

## Abstract

CircRNAs have been the focus of research in recent years. They are differentially expressed in various human tumors and can regulate oncogenes and tumor suppressor genes expression through various mechanisms. The diversity, stability, evolutionary conservatism and cell- or tissue-specific expression patterns of circRNAs also endow them with important regulatory roles in promoting or inhibiting tumor cells malignant biological behaviors progression. More interestingly, emerging studies also found that circRNAs can regulate not only other genes expression, but also their parental gene expression and thus influence tumors development. Apart from some conventional features, circRNAs have a certain specificity in the regulation of parental gene expression, with a higher proportion affecting parental gene transcription and easier translation into protein to regulate parental gene expression. CircRNAs are generally thought to be unable to produce proteins and therefore the protein-coding ability exhibited by circRNAs in regulating parental gene expression is unique and indicates that the regulatory effects of parental gene expression by circRNAs are not only a competitive binding relationship, but also a more complex molecular relationship between circRNAs and parental gene, which deserves further study. This review summarizes the molecular mechanisms of circRNAs regulating parental gene expression and their biological roles in tumorigenesis and development, aiming to provide new ideas for the clinical application of circRNAs in tumor-targeted therapy.

## Introduction

When something goes wrong in a multicellular body like the human body, normal cells will lose sense of their surroundings, cooperate with surrounding cells, start to grow uncontrollably, and gradually lose their function (1, 2). These changes eventually cause normal cells to mutate into a new type of cells (cancer cells), which can lead to impaired organ function or eventual death in the human body. Humans have always been passively resistant to cancer and scientists have tried every possible way to fight the progression of most malignant cancers rather than cure them (3). In order to overcome this disease that brings great harm to human beings, scientists have begun to explore the deep mechanism of cancer occurrence and development and subsequently discover the complexity of cancer molecular mechanisms and the randomness of tumorigenesis (4, 5). The most fundamental molecular mechanism of cancer occurrence and development can be attributed to genetic mutation, abnormal gene expression and post-translational modification of various expression products (6, 7). These processes are mainly implemented by various regulatory factors produced by human cells and more than half of them are ncRNAs (non-coding RNAs). As major regulators, ncRNAs are found to play key or auxiliary roles in various malignancies and are considered to be an important complement to the central dogma of cancer, with the potential to become targeted therapeutic drugs (8, 9).

**Figure 1:**
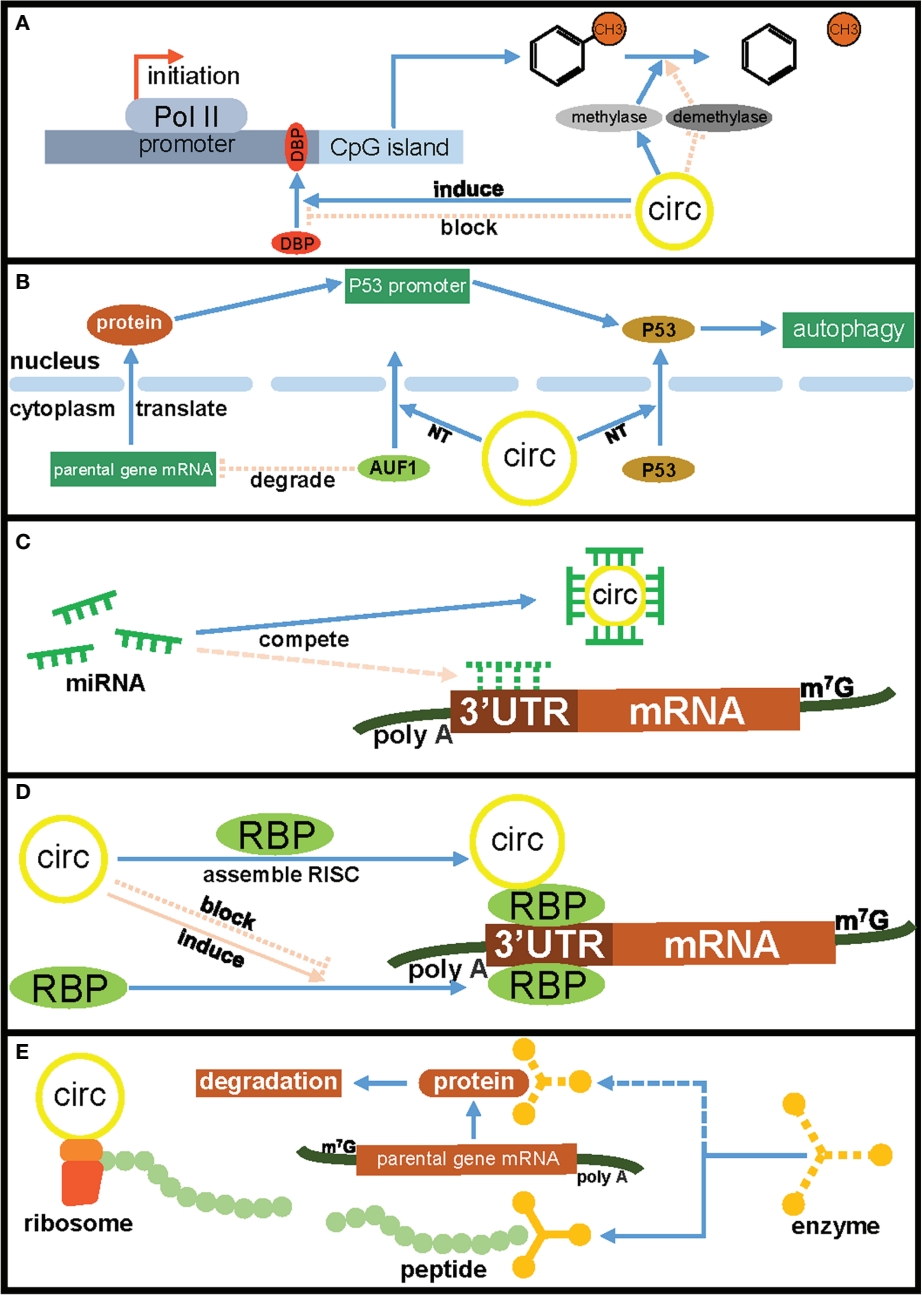
The mechanisms of circRNAs regulated parental gene expression. **(A)** CircRNAs affect parental gene transcription by competing for DNA binding proteins and influencing DNA methylation. **(B)** CircRNAs regulate parental gene expression by inducing nuclear translocation of RNA binding protein and tumor suppressor protein P53. **(C)** CircRNAs regulate parental gene expression through the ceRNA mechanism. **(D)** CircRNAs regulate parental gene expression by competing for RNA-binding proteins. **(E)** CircRNAs regulate parental genes encoded protein degradation by encoding peptides. CIRC, circRNAs; DBP, DNA binding protein; NT, nuclear translocation; RBP, RNA binding protein.

CircRNAs (circular RNAs), as a new type of ncRNAs, are becoming a new research hotspot. Unlike linear RNAs, circRNAs lack free-end structures because their 3’ and 5’ ends are covalently linked together, and this unique structure enhances their stability and protects them from RNA exonuclease mediated degradation (10, 11). In addition, circRNAs mainly exist in the cytoplasm or exosomes, and have the characteristics of tissue specificity, disease specificity, timing specificity and high conservation (12). CircRNAs have always been considered to be the product of incorrect splicing of precursor mRNA and have the ability to regulate gene expression and carry out post-translational modification (13). The emerging evidence had showed that circRNAs played important roles in various malignant biological behaviors of tumor cells, including cell proliferation, metastasis, anti-apoptosis, drug resistance and myeloid differentiation, and could affect the tumor occurrence and development *via* different molecular mechanisms including acting as ceRNA (competing endogenous RNA), RBP-binding molecules, protein translation templates and transcriptional regulators (14, 15). CircRNAs have also recently been found to regulate not only the expression of other genes, but also the expression of their parental gene through a variety of mechanisms, such as transcriptional and splicing regulation, competitive binding, translational regulation and post-translational modifications (**Table 1**), which endowing circRNAs with various important biological functions such as affecting tumor cell proliferation, metastasis, anti-apoptosis and subcutaneous transplanted tumors growth (**Table 2**). In addition, circRNAs have some functional differences in regulating the expression of parental genes and other target genes. For example, one-third of circRNAs implicated in regulating parental gene expression play inhibitory roles in tumors occurrence and development. In addition, the regulatory mechanisms of circRNAs on parental gene expression are also more complex, including promoting DNA methylation of parental gene promoter region, regulating nuclear translocation of RNA binding protein or parental gene encoded protein, and directly translating into protein to regulate parental gene expression, etc. (**Figure 1**). Studying circRNAs and their regulatory effects on parental gene expression will help to better understand the biological functions of circRNAs and deepen the exploration of the molecular mechanism of tumors occurrence and development and accelerate the application of circRNAs in the clinical treatment of malignant tumors.

**Table 1:** The mechanisms of circRNAs regulate parental gene expression.

Mechanistic classification	circRNA	Location	Mechanism	References
Transcriptional regulation	circITGA7	12q13.2	interact with TF RREB1	(16)
	circ-HuR	19p13.2	interact with TF CNBP	(17)
	circFECR1	11q24.3	promote promoter demethylation	(18)
	circ-NOTCH1	9q34.3	interact with TF myc	(19)
ceRNA	circ-AKT1	14q32.3	miR-942-5p sponge	(20)
	circFBLIM1	1p36.21	miR-346 sponge	(21)
	circ-MAPK4	18q21.1-q21.2	miR-125a-3p sponge	(22)
	circ-VANGL1	1p13.1	miR-605-3p sponge	(23)
	circTFRC	3q29	miR-107 sponge	(24)
	circ-TFF1	21q22.3	miR-326 sponge	(25)
	circ_MMP2	16q12.2	miR-136-5p sponge	(26)
	circ-ENO1	1p36.23	miR-22-3p sponge	(27)
	circYY1	14q32.2	miR-769-3p sponge	(28)
	circGFRA1	10q25.3	miR-34a sponge	(29)
	circ-EPB41L5	2q14.2	miR-19a sponge	(30)
	circ_LARP4	12q13.12	miR-513b-5p sponge	(31)
	circ-PTEN	10q23.31	miR-155/miR-330-3p sponge	(32)
	circ-ITCH	20q11.22	miR-7/miR-20a sponge	(33)
	circ-XPO1	2p15	miR-23a-3p/miR-23b-3p/miR-23c/miR-130a-5p	(34)
	circAmotl1	11q21	miR-485-5p sponge	(35)
	circ-sirt1	10q21.3	miR-132-3p/miR-212-3p sponge	(36)
	circSMO742	7q32.1	miR-338-3p sponge	(37)
Translational regulation	circ-CCND1	11q13.3	regulate parental gene mRNA stability	(38)
	circ-MMP9	20q13.12	regulate parental gene mRNA stability	(39)
	circPABPN1	14q11.2	regulate parental gene mRNA stability	(40)
	circ-DNMT1	19p13.2	regulate parental gene mRNA stability	(41)
	circSPI1	11p11.2	interact with translation initiation factor eIF4AIII	(42)
encode peptides	circ-SHPRH	6q24.3	encodes the SHPRH-146aa protein	(43)
	circFBXW7	4q31.3	encodes the FBXW7-185aa protein	(44)
	circβ-catenin	3p22.1	encodes the β-catenin-370aa protein	(45)
	circ-AKT3	1q43-q44	encodes the AKT3-174aa protein	(46)

TF, transcription factor.

**Table 2:** The role of parental genes encoded circRNAs in malignant tumors.

CircRNA	Tumor type	Dysregulation	Kaplan-Meier analysis and clinicopathological features	Biological function *in vitro*	Animal model
circITGA7	colorectal cancer	down/tumor suppressor	tumor size, lymph metastasis, distant metastasis and TNM	proliferation, migration and invasion	growth
circ-ITCH	colorectal cancer	up/oncogene	/	proliferation and Wnt/β-catenin signalling pathway	/
circFBLIM1	hepatocellular cancer	up/oncogene	/	proliferation, migration, invasion and apoptosis	growth
circ_MMP2	hepatocellular cancer	up/oncogene	poor survival	promote cells migration and invasion	/
circβ-catenin	liver cancer	up/oncogene	/	proliferation, migration and invasion	growth and lung metastasis
circGFRA1	triple-negative breast cancer	up/oncogene	tumor size, TNM, lymph node metastasis, histological grade and poor survival	proliferation and apoptosis	growth
circFBXW7	triple-negative breast cancer	down/tumor suppressor	tumor size, lymph node metastasis and favorable prognosis	proliferation, migration and invasion	growth and lung metastasis
circ-TFF1	breast cancer	up/oncogene	/	proliferation, migration, invasion and EMT	growth
circYY1	breast cancer	up/oncogene	TNM, lymph node metastasis and poor survival	proliferation, migration, invasion and glycolysis	growth
circFECR1	breast cancer	up/oncogene	/	invasion	/
circ-DNMT1	breast cancer	up/oncogene	/	proliferation and autophagy	/
circ-HuR	gastric cancer	down/tumor suppressor	/	proliferation, migration and invasion	growth and lung metastasis
circ-NOTCH1	gastric cancer	up/oncogene	poor survival	migration, invasion, tumor spheroids number and side population ratio	growth and lung metastasis
circ-sirt1	gastric cancer	down/tumor suppressor	/	proliferation, migration and invasion	growth
circ-VANGL1	bladder cancer	up/oncogene	tumor stage, lymph node metastasis and poor survival	proliferation, migration, invasion and cell cycle	growth
circTFRC	bladder cancer	up/oncogene	tumor grade, lymphatic metastasis and poor survival	proliferation, migration and invasion	growth
circ-MAPK4	glioma	up/oncogene	TNM	proliferation, migration and apoptosis	growth and the brain tumors formation
circSMO742	glioma	up/oncogene	/	proliferation, migration and apoptosis	growth
circ-SHPRH	glioma	down/tumor suppressor	/	proliferation	growth
circ-EPB41L5	glioblastoma	down/tumor suppressor	age, number of lesions, necrosis change, recurrence and poor survival	proliferation, migration and invasion	growth
circ-AKT3	glioblastoma	down/tumor suppressor	favorable prognosis	proliferation and radiation resistance	growth
circ-PTEN	non-small cell lung cancer	down/tumor suppressor	small tumor size and early TNM and favorable prognosis	proliferation	growth
circ-ENO1	lung adenocarcinoma	up/oncogene	/	proliferation, migration, EMT, glycolysis and apoptosis	growth and lung metastasis
circ-XPO1	osteosarcoma	up/oncogene	poor survival	proliferation, migration, and inhibit apoptosis	/
circ_LARP4	ovarian cancer	down/tumor suppressor	/	proliferation, migration and invasion	/
circ-CCND1	laryngeal squamous cell carcinoma	up/oncogene	larger tumor size, poor differentiation, advanced TNM and poor survival	proliferation and cell cycle	growth
circ-MMP9	oral squamous cell carcinoma	up/oncogene	TNM, lymphatic metastasis and poor survival	migration and invasion	lung metastasis
circAmotl1	cervical cancer	up/oncogene	poor survival	proliferation, migration and invasion	growth
circPABPN1	cervical cancer	down/tumor suppressor	/	proliferation	/
circ-AKT1	cervical cancer	up/oncogene	/	proliferation, migration, invasion and EMT	growth
circSPI1	acute myeloid leukemia	up/oncogene	/	proliferation, myeloid differentiation and apoptosis	/
circ-Foxo3	acute myeloid leukemia	down/tumor suppressor	poor survival	/	/

## The biogenesis and classification of circRNAs

CircRNAs were first discovered in RNA viruses in 1976, but this structurally unique closed-loop noncoding RNAs were not confirmed to exist in humans until 1993 (47, 48). When circRNAs were first discovered, they had been overlooked as the by-product of linear analog aberrant cleavage due to their closed single-stranded structure (49). With the development of transcriptome sequencing and high-throughput sequencing technology in recent years, the biological functions of circRNAs in human body and their key roles in cancer diseases had been revealed. CircRNAs were mainly formed during the processing of RNA polymerase II performing transcriptional functions according to the central dogma (50). Due to the uncertainty of the gene transcription process, the biosynthesis of circRNAs also had various mechanisms including intron pairing mediated circularization, RNA binding protein mediated circularization, alternative splicing mediated circularization (lariat-driven) and canonical linear splicing mediated circularization (intronic lariat-driven) (51). According to the splicing method and the corresponding product structure characteristics at the same time, circRNAs could be divided into three types including: exonic circRNAs (ecRNAs), exon-intron circRNAs (EIciRNAs) and intron circRNAs (ciRNAs).

## The role and molecular mechanisms of circRNAs regulating parental gene expression in tumors

CircRNAs had been shown to be important regulators in tumorigenesis and development. Recently, the crucial roles of circRNAs regulating parental gene expression in tumors were also becoming a hot spot of circRNAs research field. According to different molecular regulation mechanisms, the phenomenon of circRNAs regulating parental genes expression and their biological roles in tumors were classified here, in order to deepen the understanding of circRNAs and provide new entry points for future research.

### CircRNAs regulate parental gene transcription

Non-coding RNAs accounted for approximately 60% of the transcriptional output of human cells and had been shown to regulate a variety of cellular processes under physiological and pathological conditions (13, 52). RNA polymerase II was also required by cancer-related genes in eukaryotes nucleus to produce non-coding RNAs such as circRNAs and this synergistic process was precisely regulated by multiple factors (53). More interestingly, recent studies had found that circRNAs encoded by certain oncogenes or tumor suppressor genes could regulate the transcription process of their host genes. Li et al. found that circITGA7 or its parent gene ITGA7 (integrin subunit alpha 7) knockdown significantly enhanced proliferative and metastatic ability in colorectal cancer tumor cells *in vitro* and accelerated subcutaneous transplanted tumors growth *in vivo* (16). CircITGA7 was a negative regulator of the Ras signaling pathway and its host gene ITGA7 was associated with cytokine-related signaling pathways according to RNA-seq and KEGG (Kyoto encyclopedia of genes and genomes) enrichment analysis (54). Furthermore, the researchers found that transcription factors RREB1 (ras responsive element binding protein 1), a key regulator of Ras signaling pathway, had seven binding sites on ITGA7 gene promoter region and could inhibit ITGA7 transcription by competitively binding to ITGA7 promoter. Moreover, it was further confirmed by rescue experiments that circITGA7 could downregulate RREB1 expression through Ras pathway and thus upregulated host gene ITGA7 transcription. These results suggested that circITGA7 inhibited colorectal cancer process by suppressing the Ras signaling pathway and thus promoted tumor suppressor genes ITGA7 transcription. In addition, the study of Yang et al. found that circ-HuR could inhibit the proliferation, metastasis and microangiogenesis of gastric cancer tumor cells by silencing transcription of parent gene HuR (human antigen R), a protein stabilizing AU-rich element-containing protein (17). The further studies on the mechanism of circ-HuR regulating gastric cancer progression showed that circ-HuR could interact with CNBP (CCHC-type zinc finger nucleic acid binding protein) by binding its RGG (arg gly gly) box domain and thus regulate HuR transcriptional activity by inhibiting the binding of CNBP to HuR promoter, indicating that circ-HuR could inhibit gastric cancer progression by inhibiting parent gene HuR transcription.

DNA methylation was a DNA modification process and usually associated with transcriptional silencing. The regulatory effect of DNA methylation by circRNAs had been implicated in human tumors, but how they caused malignancy was still a mystery (55, 56). Chen et al. found that circFECR1 was transcribed from exons 4 and 2 in FLI1 (friend leukemia virus integration 1) promoter chromatin complex and confirmed that circFECR1 could promote breast cancer cell metastasis by increasing host gene FLI1 expression (18). Moreover, the binding sequence of circFECR1 to the FLI1 promoter contained abundant CpG islands. The further mechanistic studies found that circFECR1 could bind the promoter region of DNMT1 (DNA methyltransferase 1), a methyltransferase essential for maintaining DNA methylation, to downregulate DNMT1 transcription (57). The transcriptional regulatory capacity caused by DNA methylation was usually co-regulated by DNA methyltransferase and DNA demethylase, circFECR1 was also found to recruit the DNA demethylase TET1 (tet methylcytosine dioxygenase 1) in the promoter region of the host gene FLI1 to directly induce DNA demethylation of the parent gene, thereby regulating the transcriptional activity of the target gene FLI1 (58). These data suggested that circFECR1 as an upstream regulator could induced parental gene FLI1 gene transcription by coordinating the regulation of DNA methylation and demethylase to promote breast cancer progression. In addition, circ-NOTCH1 had also been found to promote gastric cancer metastasis and stemness through sponging miR-449c-5p (19). Subsequently, myc was identified as a miR-449c-5p downstream gene and could bind with the promoter region of NOTCH1 (notch receptor 1). It was also discovered that circ-NOTCH1 and its parent gene NOTCH1 expression were both increased by enhancing myc expression, while circ-NOTCH1 or NOTCH1 downregulation were found to partially reverse the promoting effect of myc overexpression in tumor cells metastasis and stemness *via* rescue assays. These results demonstrated that circ-NOTCH1 could promote host gene NOTCH1 transcription through circ-NOTCH1/miR-449c-5p/myc/NOTCH1 positive feedback loop.

### CircRNAs affect parental gene expression *via* ceRNA mechanism

MiRNAs (microRNAs) could affect tumor suppressors or oncogenes expression by binding with 3’-UTR (untranslated regions) of downstream target genes (59, 60). In the cytoplasm, a number of circRNAs had been found to act as miRNAs molecular sponges to regulate target genes expression. Considering the homolog of circRNAs with parental gene in tumors, it was possible that circRNAs could serve as competing endogenous RNA to regulate their parental gene expression and thus promote tumors occurrence and development (61). For example, upregulated circ-AKT1 could promote its parental gene AKT1 (v-akt murine thymoma viral oncogene homolog 1) expression by acting as miR-942-5p molecular sponge to suppress ovarian cancer tumor cells proliferation and migration (20). The apoptosis and cell cycle of tumor cell played important roles in tumor growth (62), recent studies had found that circFBLIM1 could function as a ceRNA to upregulate host gene FBLIM1 expression through sponging miR-346 and thus exert inhibitory effect in tumor cells apoptosis in hepatocellular cancer; circ-MAPK4 silencing had also been confirmed to downregulate its parental gene expression through ceRNA mechanism, resulting in upregulated phosphorylation level of P38/MAPK (mitogen-activated protein kinase) in glioma tumor cells and promoting tumor cells apoptosis (21, 22). In addition, circ-VANGL1 knockdown was also found to prevent tumor cell cycle progression, resulting in more cells blocked at the G0/G1 stage (23). Metastatic ability was a key hallmark of cancer progression, and this characteristic could be achieved through epithelial-mesenchymal transition, circTFRC could inhibit miR-107 expression to facilitate downstream host gene expression and thus promote epithelial-mesenchymal transition in bladder cancer metastatic progression (24, 63). Moreover, this ability was also found in circ-TFF1, which could induce epithelial-mesenchymal transition and therefore promote breast cancer tumor cells metastasis through the ceRNA mechanism (25). Exosomes were also drivers for tumor cells metastatic spread, circ_MMP2 was found to be driven by high metastatic tumor cells-secreted exosomes into normal hepatocellular cells and promoted tumorigenesis and metastasis in hepatocellular cancer *via* sponging miR-136-5p to enhance parental gene MMP2 (matrix metalloproteinases 2) expression (26, 64). More notably, matrix metalloproteinases were important driving force for extracellular matrix and tissue destruction during tumor invasion and metastasis (65). In addition, recent study also reported that circ-ENO1 knockdown could cause a reduction on glycolytic enzyme ENO1 (enolase 1) expression and thus reduced ATP production, delayed the relative glucose uptake as well as decreased the lactate production (27). This effect was also found in breast cancer, while circYY1 could affect glycolysis process by regulating parental gene YY1 (yin yang 1) expression by acting as miR-769-3p molecular sponge (28). Due to the existence of tumor microenvironment and its many regulatory factors, it was one of circRNAs research frontiers to study whether there are differences in the effects of circRNAs regulating tumors development *in vivo* and *in vitro* (66). The several groups of researchers had confirmed that circGFRA1, circ-EPB41L5 and circ_LARP4 could promote tumor growth and lung metastasis in nude mice by affecting their homologous host gene expression through acting as miRNAs molecular sponges (29–31). Moreover, some biological factors in mice could also help circRNAs to play carcinogenic or anticancer roles. For example, the presence of bax, cleaved caspase-3, MMP2 and other substances was the material basis for circ_LARP4 to promote tumor growth and lung metastasis by upregulating parental gene expression.

Through the usage of high-throughput RNA sequencing, a large number of circRNAs were found to be able to regulate cancer-related signaling pathways by affecting host gene expressions such as, circ-PTEN increased its particular parental gene PTEN (phosphatase and tensin homolog) expression by acting as the molecular sponge for miR-155 and miR-330-3p, resulting in non-small cell lung cancer cells growth inhibition and the inactivation of the carcinogenic PI3K/AKT signalling pathway, an important regulatory pathway of signalling and intracellular vesicular trafficking (32, 67). In addition, circ-ITCH also played an important inhibitory role in colorectal cancer through functioning in the Wnt/β-catenin pathway by affecting host gene expression and Wnt/β-catenin pathway had key regulatory role in many tumor malignant biological behaviors such as cell proliferation, metastasis and epithelial-mesenchymal transition (33, 68). In addition, the researchers constructed the ceRNA working network and found that circRNAs could regulate host genes expression through multiple regulatory pathways such as, circ-XPO1 could sponge miR-23a-3p, miR-23b-3p, miR-23c and miR-130a-5p to regulate parental gene XPO1 (exportin 1) expression (34). Whether multiple miRNAs acting as indispensable mediators between circRNAs, and their parental gene could maintain the stability and adequacy of circRNAs’ functions deserved further study. Besides the above, circRNAs including circAmotl1, circ-sirt1 and circSMO742 had been found to exert function as miRNAs molecular sponges and could regulate their cancer-related parental gene expression to affect human cancers including gastric cancer, cervical cancer and glioblastoma progression (35–37).

### CircRNAs affect mRNA stability to regulate parental gene expression

The emergence and refinement of central dogma in molecular biology, for the first time, systematically and correctly explained the transmission of genetic information from DNA to functional products such as proteins (69, 70). In some cases, mRNA concentration was highly linear with functional products and the regulatory of mRNA stability and translation ability could affect the production of functional products, especially proteins (71). The untranslated regions of ncRNAs such as circRNAs could form large base pairs with complex structures and these base pairs had been found to regulate mRNA stability by binding to RNA-binding proteins or interacting directly with the secondary and tertiary structures of mRNA (72, 73). CircRNAs were involved in the whole process of gene expression and the regulatory effect of circRNAs on mRNA stability were important supplementary pathway for circRNAs to affect gene expression regulation. The regulatory effect of circRNAs on their host gene expression was also involved in mRNA stability. Circ-CCND1 and its host gene CCND1 (cyclin D1) were found to be highly expressed in laryngeal squamous cell carcinoma and could promote tumor cells proliferation and metastasis (38). In order to further study the carcinogenic mechanism of circ-CCND1 regulating parental gene expression, the researcher tested CCND1 mRNA stability by blocking *de novo* transcription in cells treated with actinomycin D and result data showed that the half-life of CCND1 mRNA was reduced from 5.5 hours to 3 hours after circ-CCND1 knockdown. RNA pull-down assay data revealed that circ-CCND1 probe enriched a large amount of RNA binding protein HuR. Subsequently, HuR was predicted to bind with 83-134bp of circ-CCND1 *via* online catRAPID algorithm. Moreover, the RNA pull-down assay was again used and confirmed that the mutation in 83-134bp region of circ-CCND1 could block the interaction between circ-CCND1 and HuR. It was disclosed that ectopic HuR expression could partially reverse the decreased mRNA level of CCND1 mediated by circ-CCND1 knockdown. The regulatory effect of HuR protein to mRNA largely depended on its special molecular structure. HuR protein consisted of three RNA recognition motifs (RRM) and a hinge region. RRM1 and RRM2 at the N-terminal could bind with au-rich elements (AREs) of the 3’-untranslated region of targeted mRNA and AREs could accelerate the degradation of the 3’-untranslated region to break mRNA stability (74, 75). These results suggested that circ-CCND1 might block the degradation of AREs by recruiting HuR protein in the CCND1 3’-UTR region and therefore enhanced the stability of CCND1 mRNA, promoting laryngeal cancer progression. Accumulating evidence suggested that MMP9 played a key positive role in OSCC (oral squamous cell carcinoma) tumor cells migration and invasion across the basement membrane (39). Clinicopathological data analysis showed that patients with high circ-MMP9 expression were more likely to have lymph node metastasis and advanced TNM stage. Functionally, circ-MMP9 knockdown could weaken the metastasic and invasive abilities of OSCC tumor cells *in vitro* and inhibited lung metastasis *in vivo*. Mechanistically, circ-MMP9 was found to block the regulatory effect of AUF1 (au-rich element-RNA binding factor) on the matrix metalloproteinase 9 3’-untranslated region. Different from the HuR described above, AUF1 could bind to mRNAs containing ARE and then assemble to form a multi-subunit complex to directly and rapidly degrade the mRNA substrate. These results suggested that circ-MMP9 could enhance parent gene expression by blocking the degradation of AUF1 to MMP9 mRNA, thereby promoting the metastasis of OSCC tumor cells.

The regulatory effect of circRNAs on the mRNA stability of parental genes also showed multiplicity and this might be associated with persistent dysregulated expression of some genes. CircRNAs not only promoted the translation of parent genes, but also reduced the expression of host genes by preventing the mRNA stability improvement mediated by RNA-binding proteins (76). Some researchers found that HuR didn’t affect circPABPN1 expression in cervical cancer, but increased circPABPN1 could inhibit the binding of HuR to PABPN1 (poly(A) binding protein nuclear 1) mRNA (40). RIP and qRT-PCR analysis also showed that the binding of HuR to multiple target mRNAs (including PABPN1 mRNA) was significantly inhibited in tumor cells with higher circPABPN1 expression. Polymorphism analysis of PABPN1 mRNA showed that overexpressed circPABPN1 significantly inhibited parental gene PABPN1 translation and upregulated HuR could partially reverse this effect. CircPABPN1 overexpression could decrease mRNA level of parental gene PABPN1, while HuR overexpression upregulated PABPN1 mRNA level. Representative sucrose gradient profiles confirmed that HuR could bind with PABPN1 mRNA and promote its translation, while circPABPN1 competitively binding HuR to block its binding to PABPN1 mRNA, thereby inhibiting the translation of parental gene PABPN1. These results suggested that circPABPN1 could promote host gene PABPN1 mRNA degradation by competitively binding HuR, thus inhibiting parental gene translation and playing a tumor suppressor role in cervical cancer.

Nuclear Translocation was a subcellular process and modified cell function primarily by transporting active cytoplasmic proteins into the nucleus, and the regulatory effect of circRNAs on parental gene expression was also involved in this particular mechanism (77). Du et al. found that circ-DNMT1 expression was elevated in eight breast cancer tumor cell lines and patients with breast cancer, and highly expressed circ-DNMT1 could increase the proliferation and viability of breast cancer tumor cells by stimulating autophagy (41). Moreover, circ-DNMT1 was found to elevate the parental gene DNMT1 expression and the highly expressed DNMT1 could be transferred from the cytoplasm to the nucleus. The further mechanistic studies found that circ-DNMT1 could interact with p53 and AUF1 to promote the nuclear translocation of these two proteins. Subsequent studies found that nuclear translocation of AUF1 induced by circ-DNMT1 could reduce the parental gene DNMT1 mRNA degradation in the cytoplasm and the increased parental gene DNMT1 expression could enter the nucleus to inhibit p53 transcription. These results suggested that circ-DNMT1 could block the degradation of the parental gene DNMT mRNA by inducing AUF1 nuclear translocation and then inhibit the transcription of nuclear p53 through the nuclear translocation of DNMT, thereby affecting tumor cell autophagy.

The special phenomenon of circRNAs regulating parental genes expression was also present in hematological tumors. Wang et al. found that circSPI1 was significantly overexpressed in acute myeloid leukemia (AML), while its parental gene SPI1 (salmonella pathogenicity island 1) was lowly expressed. Moreover, circSPI1 and its host genes SPI1 had opposite roles in causing AML tumor cell proliferation, myeloid differentiation and apoptosis. In the rescue experiment, circSPI1 overexpression could antagonize the inhibitory effect of its parental gene-encoded protein PU.1 (purine-rich nucleic acid binding protein 1) on the AML malignant biological behavior progression. The mechanism research found that circSPI1 and SPI1 co-regulated 223 differentially expressed genes, and circSPI1 could antagonize PU.1 protein expression by interacting with the translation initiation factor eIF4AIII. These results suggested that circSPI1 might regulate the abnormal expression of many genes by regulating the translation process of the parental gene SPI1, thereby affecting AML occurrence and development (42). In addition, another group of researchers found that circ-Foxo3 and its parental gene Foxo3 (forkhead box O3) was lowly expressed in AML tumor cells than that in bone marrow nucleated cells, and the overall survival time of AML patients with high Foxo3 expression was longer than those with low Foxo3 expression. The spearman correlation test results showed that there was a significant positive correlation between circ-Foxo3 and its parental gene Foxo3 expression in AML patients. The multivariate analysis showed that karyotype classification, Foxo3 expression and patient age were prognostic factors for AML. Among them, Foxo3 expression was a protective factor, and karyotype classification and age were poor prognostic factors. These results suggested that circ-Foxo3 might play a tumor suppressor role in AML by regulating parental genes expression, and the specific mechanism of their effect was also worthy of further study (78).

### CircRNAs regulates parental gene expression by encoding peptides

Precursor proteins were inactive and often accepted a series of post-translational processes before becoming functional mature proteins (79). In some cases, the translation products of circRNAs often had partially identical amino acid sequences with the proteins encoded by the parental genes (80). Therefore, circRNAs and parental gene encoded protein might be competitively bound to the same enzymes or other regulatory molecules, endowing circRNAs the ability to affect the degradation of the protein translated by the parent gene. Zhang et al. found that circ-SHPRH with an IRES (internal ribosomal entry site) driven open reading frame was able to translate a new functional protein called SHPRH-146aa (43). Moreover, circ-SHPRH and SHPRH-146aa were lowly expressed in glioblastoma and overexpressed SHPRH-146aa could increase parental gene SHPRH (SNF2 histone linker PHD RING helicase) expression to downregulate its tumorigenicity *in vitro* and *in vivo*. According to previous reports, full-length SHPRH protein could specifically mediate the ubiquitination degradation of PCNA and promote tumor cells malignant biological progression, while E3 ubiquitin ligase DTL (dentideless) could prevent PCNA degradation by competitively binding to full-length SHPRH. Further research found that circ-SHPRH could increase full-length SHPRH protein expression without affecting its mRNA expression after transfection of circ-SHPRH overexpression vector. Mechanismly, SHPRH-146aa contained two SHPRH ubiquitination sites K1562 and K1572, and overexpressed SHPRH-146aa could competitively bind to E3 ligase DTL to protect SHPRH from ubiquitination (81). Based on the above information, it could be inferred that the SHPRH-146aa protein produced by circ-SHPRH could protect the full-length SHPRH from DTL-induced ubiquitination, thereby inducing PCNA (proliferating cell nuclear antigen) degradation to inhibit glioblastoma progression. Ubiquitin-proteasome pathway was a common endogenous protein degradation pathway. The protein was firstly modified by ubiquitination and then degraded by the proteasome (82). CircFBXW7 was also found to encode the FBXW7-185aa protein to affect parental gene FBXW7 (F-box and WD repeat domain containing 7) protein level and upregulated FBXW7-185aa inhibited proliferation and cell cycle acceleration in triple-negative breast cancer cells, while downregulated FBXW7-185aa promoted malignant phenotypes *in vitro* and *in vivo* (44). Western blot analysis showed that overexpressed FBXW7-185aa increased FBXW7 abundance and induced c-Myc degradation, while transfection of USP28 decreased FBXW7 abundance and suppressed FBXW7-185aa induced c-Myc destabilization. Mechanistic studies found that FBXW7-185aa inhibited the binding of USP28 (ubiquitin specific peptidase 28) to FBXW7 by competitively binding to USP28, and thus elevating protein level of parental gene FBXW7, and overexpressed FBXW7 reduced c-MYC stability. These results suggested that the FBXW7-185aa protein encoded by circ-FBXW7 could inhibit ubiquitination of the parent gene protein by competitively binding USP28, thus downregulating c-MYC expression and inhibiting the occurrence and development of breast cancer. The abnormal activation of Wnt/β-catenin signaling pathways was closely associated with increased prevalence, malignant progression, development of poor prognosis, and even increased cancer-related mortality (45). Liang et al. found that circβ-catenin encoded by host gene β-catenin was highly expressed at liver cancer tumor tissues and cells, and circβ-catenin knockdown could inhibit tumor cell growth, migration and cell cycle progression (83). The further mechanistic studies found that a novel β-catenin isoform named β-catenin-370aa encoded by circβ-catenin could physically interact with GSK3β (glycogen synthase kinase-3 beta), an upstream regulatory node of the ubiquitin-proteasome pathway (84). Some previous studies had confirmed that the β-catenin protein stability was closely linked with its phosphorylation status (46). After phosphorylation by GSK3β, the residues of β-catenin protein such as Ser33, Ser37 and Thr41 were ubiquitinated by the ubiquitin ligase β-TrCP and subsequently degraded by the proteasome. The co-IP results assay showed that silencing of circβ-catenin enhanced the interaction between GSK3β and full-length β-catenin, while downregulating the expression of β-catenin-370aa partially blocked the interaction between GSK3β and full-length β-catenin. Co-IP also found that β-catenin ubiquitination levels were increased after knockdown of circβ-catenin, suggesting that circβ-catenin may enhance β-catenin stability. These results suggested that β-catenin-370aa encoded by circβ-catenin could competitively bind GSK3β, thereby preventing ubiquitination-mediated parental gene β-catenin protein degradation and promoting the malignant biological behaviors of liver cancer.

AKT, also known as protein kinase B (PKB), was a 60kDa silk/threonine protein kinase that played an important role in tumor progression by acting as the initiating node for related signaling pathways (85, 86). Circ-AKT3, a transcription variant from parental gene AKT3, had been found to be lowly expressed in glioblastoma tumor tissues and could encode a 174 amino acid novel protein named AKT3-174aa by utilizing overlapping start-stop codons. *In vitro* and *in vivo* experiments proved that AKT3-174aa, but not circ-AKT3, exerted a tumor-suppressive role in glioblastoma. Activated PDK1 could phosphorylate the exposed thr308 site to fully activate AKT. Mass spectrometry (MS) found that p-PDK1 protein was predicted to interact with AKT3-174aa. Immunofluorescence staining of glioblastoma cells transfected with Flag-tagged AKT3-174aa also supported the co-localization of AKT3-174aa with p-PDK1, suggesting that AKT3-174aa preferred to inhibit thr308 of AKT by interacting with p-PDK1. These results demonstrated that Akt3-174aa could interact with phosphorylated inactive PDK1 to reduce the phosphorylation of Akt-Thr308, thus playing a tumor-suppressive role in glioblastoma.

## Conclusion

Since the research on circRNAs is still in its infancy, the functions of circRNAs molecules are not well understood. Advances in the field of circRNAs research have revealed the biological origin of circRNAs and their important roles in the deep molecular mechanisms of tumorigenesis and development, endowing circRNAs with the potential to become next-generation targeted drugs for tumor therapy (87, 88). However, there are still many challenges on the way to the use of circRNAs as targeted therapeutics, and the most important one is the uncertainty about the molecular mechanism of circRNAs function in tumors (89). Currently, circRNAs typically regulate tumor malignant biological behaviors are mainly as miRNAs sponges, transcriptional regulators, protein decoys/scaffolds and protein translation templates. However, there are also some functional classifications of circRNAs regard the regulatory effect of circRNAs on parental gene expression as a separate function, because the regulatory effect of circRNAs on parental gene expression is different from that on other target genes. For example, circRNAs can also inhibit the degradation of proteins encoded by parental gene, which are rarely found in the regulation effects of circRNAs on other target genes. Moreover, the phenomenon that the circRNAs transcribed by the host gene can in turn regulate the expression of the parental gene is unique in itself and deserves further study whether the occurrence of this regulatory effect is accidental or inevitable. Some general functions of circRNAs, such as miRNAs molecular sponges and protein scaffolds, have been thoroughly studied in previous studies and the research on circRNAs needs to move towards deeper characteristics. With the deeper exploration of the functional mechanism of circRNAs, it will help to better understand the pathological process of tumorigenesis and development and pave the way for future circRNA-based tumor diagnosis and therapeutic intervention.

## Author contributions

HW, XG, SY, and WW wrote the manuscript and prepared the tables. GL collected relevant data. XJ and DS are in charge of project administration and funding acquisition. All authors contributed to the article and approved the submitted version.

## Funding

This work was supported by grants from Heilongjiang Postdoctoral Science Foundation (LBH-Q21023), National Natural Science Foundation of Heilongjiang Province (LH2020H058), Chen Xiaoping Foundation for the Development of Science and Technology of Hubei Province (CXPJJH12000002-2020015).

## Conflict of interest

The authors declare that the research was conducted in the absence of any commercial or financial relationships that could be construed as a potential conflict of interest.

## Publisher’s note

All claims expressed in this article are solely those of the authors and do not necessarily represent those of their affiliated organizations, or those of the publisher, the editors and the reviewers. Any product that may be evaluated in this article, or claim that may be made by its manufacturer, is not guaranteed or endorsed by the publisher.
